# *Candida albicans—*The Virulence Factors and Clinical Manifestations of Infection

**DOI:** 10.3390/jof7020079

**Published:** 2021-01-22

**Authors:** Jasminka Talapko, Martina Juzbašić, Tatjana Matijević, Emina Pustijanac, Sanja Bekić, Ivan Kotris, Ivana Škrlec

**Affiliations:** 1 Faculty of Dental Medicine and Health, Josip Juraj Strossmayer University of Osijek, HR-31000 Osijek, Croatia; jtalapko@fdmz.hr (J.T.); martina.juzbasic@fdmz.hr (M.J.); 2 Department of Dermatology and Venereology, Clinical Hospital Center Osijek, HR-31000 Osijek, Croatia; tatjana.7.kovacevic@gmail.com; 3 Faculty of Natural Sciences, Juraj Dobrila University of Pula, HR-52100 Pula, Croatia; emina.pustijanac@unipu.hr; 4 Family Medicine Practice, HR-31000 Osijek, Croatia; sbekic@mefos.hr; 5 Faculty of Medicine, Josip Juraj Strossmayer University of Osijek, HR-31000 Osijek, Croatia; 6 Department of Internal Medicine, General County Hospital Vukovar, HR-3200 Vukovar, Croatia; ikotris@mefos.hr

**Keywords:** *Candida albicans*, virulence traits, oral cavity, gastroenterology, dermatovenerology

## Abstract

*Candida albicans* is a common commensal fungus that colonizes the oropharyngeal cavity, gastrointestinal and vaginal tract, and healthy individuals’ skin. In 50% of the population, *C. albicans* is part of the normal flora of the microbiota. The various clinical manifestations of *Candida* species range from localized, superficial mucocutaneous disorders to invasive diseases that involve multiple organ systems and are life-threatening. From systemic and local to hereditary and environmental, diverse factors lead to disturbances in *Candida*’s normal homeostasis, resulting in a transition from normal flora to pathogenic and opportunistic infections. The transition in the pathophysiology of the onset and progression of infection is also influenced by *Candida*’s virulence traits that lead to the development of candidiasis. Oral candidiasis has a wide range of clinical manifestations, divided into primary and secondary candidiasis. The main supply of *C. albicans* in the body is located in the gastrointestinal tract, and the development of infections occurs due to dysbiosis of the residential microbiota, immune dysfunction, and damage to the muco-intestinal barrier. The presence of *C. albicans* in the blood is associated with candidemia–invasive *Candida* infections. The commensal relationship exists as long as there is a balance between the host immune system and the virulence factors of *C. albicans*. This paper presents the virulence traits of *Candida albicans* and clinical manifestations of specific candidiasis.

## 1. Introduction

### 1.1. Fungal Infection

Pathogens that causes fungal infections, such as *Candida albicans*, are widespread and may affect the skin and mucosal surface, and may cause systemic infection. Species of *Candida* are present in as many as 400,000 systemic fungal diseases [1]. Of all the species, *Candida albicans* is the most common causative agent of mucosal infections and systemic infection, and it is responsible for about 70% of fungal infections around the world [2]. It has been the leading cause of life-threatening invasive infections for the past several decades. Despite treatment, the mortality rate is close to 40%, especially in hospital conditions [3,4]. The present review aims to provide an overview of the virulence traits of *Candida albicans* and its clinical manifestations in the oral cavity, intestinal mucosa, skin, as well as in invasive infections.

### 1.2. Candida Albicans

**Figure 1:**
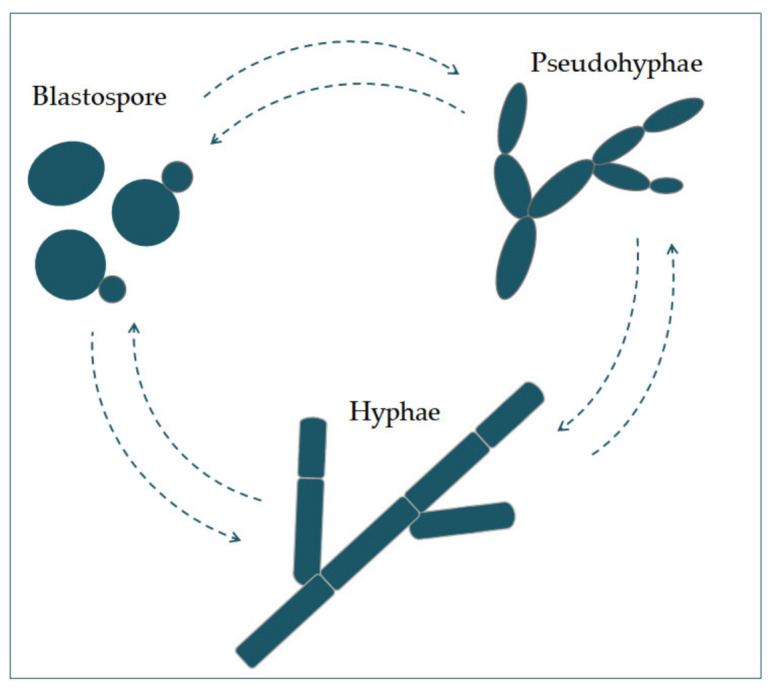
The morphological switches and transitions of *Candida albicans* during the infection process. The morphological transitions from blastospore to pseudohyphae and hyphae are reversible.

*Candida albicans* appears in several morphological forms (blastospores, pseudohyphae, and hyphae) (Figure 1). Blastospores divide asexually by budding [5,6]. During that process, new cell material is formed on the surface of the blastospore. The new bud grows from a small selected blastospore, and it is most often located distally from the site of a scar caused by birth, after which the phase of growth begins. After the growth phase ends, the cells divide, whereby the daughter separates from the parent cell by creating a partition [6].

Chains of elongated yeast cells characterize pseudohyphae, and the shape of hyphae is characterized by branched chains of tubular cells, with no narrowing at the sites of septation [7]. Filamentation is enhanced by a temperature higher than 37 °C, an alkali pH, serum, and high concentrations of CO_2_ [8]. In the same way, it is also enhanced by a lack of nitrogen and carbon in the presence of N-acetylglucosamine (GlcNAc) [7]. This transition from a blastospore to a hypha is characterized by the activation of a complex regulatory network of signal paths, which include many transcription factors [8]. The main difference between yeast and hyphae composition is that the hypha wall has slightly more chitin content than yeast [9].

The cell wall is made of glucan, chitin, and protein. Its role is to protect the cell from stressful conditions in the environment, such as osmotic changes, dehydration, and temperature changes, and protect the cells from the host’s immune defense [10,11]. It is also responsible for adhesion to the host cell, with adhesion proteins such as Als1-7, Als9, and Hwp1 [12].

Communication of the cell with the outside environment takes place through the cell membrane [13]. Sterols in the cell membrane are extremely important, giving the cell stability, rigidity, and resistance to physical stressors [9]. Ergosterol is the most represented sterol and is characteristic for the cell membrane of fungi. It is synthesized on the endoplasmic reticulum and lipid bodies [14]. In the cell membrane, there is a phospholipid bilayer containing proteins with the role of receptors, but also some whose role is transport and also signal transduction [15].

In its metabolism, *Candida albicans* uses glucose as a source of carbon and amino acids as nitrogen sources [16].

## 2. Virulence Factors of *Candida albicans*

*Candida* participates actively in the pathophysiology of the occurrence and advance of infection, thanks to its virulence factors. One group of virulence factors causes colonization to take place, or the initiation of an infection, whilst the other group helps to spread the infection [17].

**Figure 2:**
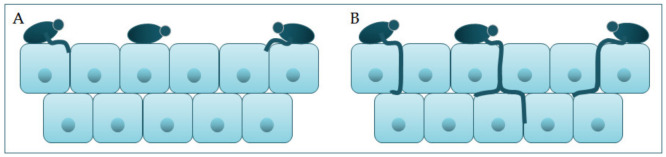
Schematic presentation of (**A**) adherence and colonization, and (**B**) penetration and invasion of *C. albicans*.

Polymorphism implies the transition of *C. albicans* from a commensal form to a pathological one, which depends on changes in the environment in which it is located. It is characterized by the morphological transition of blastospores into hyphae, and the transitional form between are pseudohyphae [18,19]. Asexual spores, chlamydospores, are formed under adverse conditions and are three to four times bigger than blastospores [12]. The morphological transition of *C. albicans* begins with the budding of blastospores and the formation of new cells. The nuclei separate at the mother–daughter cell junction via the septum [20]. The nuclei of hyphal forms divide in the germ tube but outside the septum region. After division, one nucleus returns to the mother cell, and the other moves toward the center of the germ tube [21]. *C. albicans* is present in the form of yeast in the human microbiome. The transition from yeast to the hyphal form is a transition into a pathogenic form [22,23]. The hyphal form is invasive, and in this form, the cells enter the host tissue by active penetration and induced endocytosis [24] (Figure 2). Induced endocytosis is mediated by hyphae invasion and depends on host activity, whereas active penetration depends on the fungal activity [25]. Several signaling pathways are involved in hyphal formation. The most important is cAMP-dependent protein kinase A (cyclic adenosine monophosphate PKA) [3,26].

It has been shown that a hypha-specific toxin, candidalysin, is crucial for the occurrence of candidiasis [19,27]. Candidalysin is a cytolytic 31-amino acid α-helical amphipathic peptide [19,28]. It is produced by the *C. albicans* hyphae, and it is crucial in damaging the host cells. It is thought that it contributes to establishing a systemic infection and mortality [29]. Candidalysin is capable of directly damaging the epithelial membrane, by intercalation, permeabilization, and creating pores, causing the cytoplasmic contents to weaken [29,30].

Factors that contribute to the pathogenic potential of *Candida albicans* are the expression of proteins important for adhesion and invasion. The process of adhesion is affected by various factors, such as the types of protein in the cell wall, and the physical and chemical properties of the cell surface. Adhesins of *C. albicans* recognize ligands such as proteins, fibrinogens, and fibronectins and bind to them [17]. Since adhesins such as Als3 and Hwp1 are mainly expressed during hyphae creation, they play an important role in the adhesion of *C. albicans* to the host cells [17].

**Figure 3:**
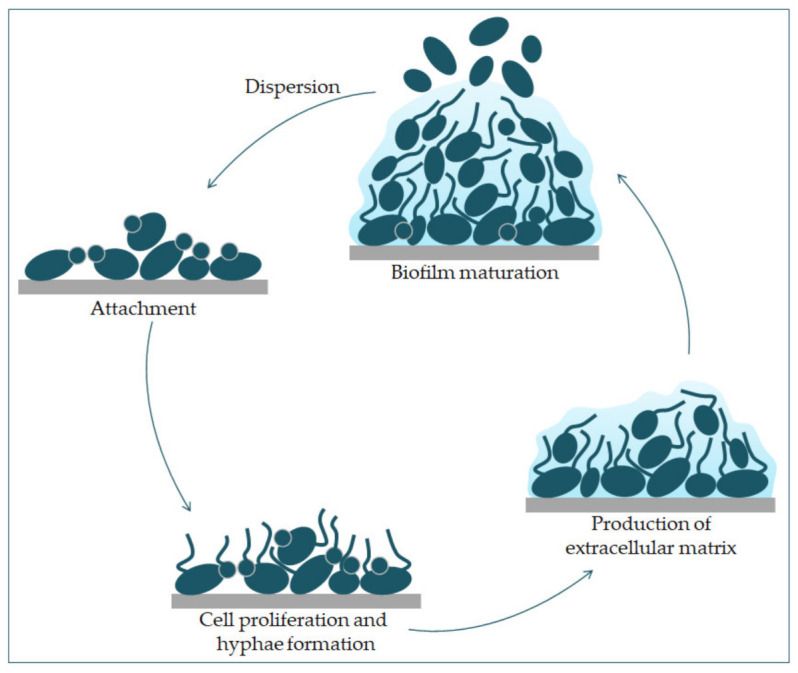
Phases of *C. albicans* biofilm formation. The formation starts with the attachment of yeast cells (green) to the surface (grey). In the early phase of the biofilm occurs the proliferation of *C. albicans* and hyphal cells’ formation. The production of the extracellular matrix follows. The maturation phase includes the accumulation of an extracellular matrix. Finally, yeast cells disperse to a new site and form a new biofilm.

Formation of biofilm is a property of *C. albicans *pathogenesis. Most infections caused by *C. albicans* are related to the creation of a biofilm on the surface of the host or on abiotic surfaces (implants), which leads to high morbidity and mortality [23]. Because *C. albicans* can transition from yeast to hyphae morphologically, its biofilm is a complex structure of different morphological forms [31]. The biofilm develops through several consecutive phases [32]. In the first phase, the individual cells of *Candida albicans* adhere to the substrate, which forms the basal layer of the biofilm. After that comes the phase of cell proliferation and filamentation, in which the cells form elongated protrusions, which continue growing into filamentous hyphal forms. The production of hyphae is a sign of the initiation of the creation of the biofilm. In the maturation phase, the accumulation of an extracellular polysaccharide matrix follows. The final phase involves the dispersion of non-adherent cells, which results in the possibility of the inception of new biofilms (Figure 3) and the possibility of dissemination in the tissue [33,34].

The extracellular polysaccharide matrix comprises extracellular polymers and extracellular DNA involved in maintaining the biofilm structure [35]. Additionally, extracellular DNA plays a vital role in binding the biofilm to the substrate [32]. An essential part of the extracellular matrix are β-1,3-glucans, which significantly contribute to the biofilm’s resistance to antifungal drugs because they prevent contact with target cells [36]. *C. albicans* cells in biofilm release more β-1,3-glucans into the extracellular matrix than planktonic cells [37]. The biofilm channels facilitate cell supply with nutrients, air, and water, giving it new “multicellular” properties [32]. Intercellular communication, or quorum sensing, is an essential factor in forming biofilm and is based on microorganisms’ behavior and the synthesis of signal molecules [38]. “Autoinducers” are signal molecules that regulate the population density by a signal mechanism. The binding of signal molecules to receptors suppresses target genes when a specific biofilm density is reached at a critical autoinducers concentration. This modulation of the quorum sensing process maintains the biofilm fungal colony’s optimal size and encodes virulent phenotypes [32]. The transcription network that regulates biofilm formation consists of six major transcription regulators (Efg1, Tec1, Bcr1, Ndt80, Rob1, and Brg1) that regulate the expression of 1000 genes [39,40]. Bcr1 transcription factor (Biofilm and Cell wall Regulator 1), whose main target is Hwp1 (Hyphal Wall Protein), is necessary to form biofilm on mucosal surfaces [41]. The Hwp1 protein binds to transglutaminases on host cells in biofilms on mucosal surfaces. While on abiotic surfaces, it is expressed as an independent enzyme of the host and has an adhesion function [42]. Several different gene products control biofilm development on abiotic surfaces transcription factors (Efg1, Bcr1, Tye7), cell wall proteins (Hwp1, Als3), protein kinases (Ire1, Cbk1) [43]. The two essential regulators of biofilm on abiotic surfaces are Efg1 and Bcr1. These transcription factors are needed for the expression of different genes for cell adhesion and filamentation in biofilms on abiotic surfaces. Additionally, the adhesin Als3 which is the target of Bcr1 plays a crucial role in the formation of biofilm on the abiotic surface [43]. During the formation of a biofilm, besides the change in expression of genes directly involved in its formation, the expression of genes indirectly related to different characteristics of the biofilm also changes [44]. The expression of genes involved in the metabolism of sulfur-containing amino acids is increased, which is characteristic of cells in the biofilm’s deeper layers. This metabolism allows cells to survive starvation and oxidative stress because sulfur amino acids are involved in the synthesis of antioxidants. The biofilm cells form a hypoxic environment and increase the expression of genes involved in glycolysis, fatty acid metabolism, and ergosterol synthesis [45].

Thigmotropism of the hyphae of *C. albicans* is regulated by the extracellular intake of calcium through calcium channels. It is an important mechanism in the enhancement of the virulence of *Candida spp.* Thigmotropism aids in creating a biofilm on abiotic surfaces and the spread in the host tissue [16].

Among virulence factors of *C. albicans *is phenotype transition between white and opaque cells. Phenotype diversity provides a quick response to changes in the environment. It is extremely important for the life of many microbe species. In *Candida albicans* cells, switching between two phenotype states, white and opaque, leads to differences in filamentous growth and interactions with immunological cells in vitro [46]. Morphological changes and phenotypic switches are stabilized transcriptionally and are stable for many generations [47].

Secretion of hydrolytic enzymes are present in *Candida albicans.* Hydrolytic enzymes facilitate the commensal and pathogenic characteristics such as attachment to host tissue and causing the host cell membrane’s rupture. Because of these enzymes, invasion into the surfaces of mucous membrane and blood vessels is possible, and they also participate in avoiding the host’s immune response. The three main enzymes produced by *C. albicans* are SAP (secreted aspartyl protease), phospholipase, and hemolysin [48].

## 3. Clinical Manifestations of Candidiasis

*Candida albicans* is part of the normal microbiota in about 50% of individuals [49]. *Candida sp*. infections have various clinical manifestations, from superficial mucocutaneous disorders to an invasive infection affecting multiple organs [50]. We present here the clinical manifestations of *C. albicans* in the oral cavity, intestinal mucosa, skin, as well as in invasive infections.

### 3.1. Candida albicans in the Oral Cavity

Candidosis or candidiasis is the most common fungal infection in the oral cavity and is caused by *Candida* species. It was previously thought that 35%–80% of the population are carriers of oral *Candida*. Recent research using molecular detection methods suggests that *Candida spp.* are found in all humans as part of the normal oral flora [51,52]. The most common species in infected and healthy mouths is *Candida albicans*, and it is estimated to be found in over 80% of oral fungal isolates. Other types of *Candida*, the so-called non-*albicans Candida* species present in the mouth are *C. glabrata*, *C. dubliniensis*, *C. parapsilosis*, *C. krusei*, and *C. tropicalis* [51,53,54,55].

Various systemic, local, hereditary, and environmental factors lead to disturbances in oral homeostasis. Consequently, the transition of the normal flora to the pathogen and an opportunistic infection occurs. The changes lead either to excessive *Candida* growth or to a change in the expression of its virulence factors [51,56]. The most common local predisposing factors for candidosis are poor oral hygiene, wearing mobile prosthetic replacements, orthodontic appliances, and obturators, dry mouth (xerostomia), smoking, and steroid inhalers use, a diet rich in carbohydrates, and diseases of the oral mucosa. The systemic predisposing factors described are age (risk groups are the elderly and newborns), pregnancy, antibiotic therapy, systemic corticosteroid therapy, diseases such as tumors and their therapy, diseases of the digestive system, nutritional deficiencies (iron, folic acid, and vitamin deficiency), endocrinopathy (diabetes, hypothyroidism, hypoparathyroidism, etc.), autoimmune diseases (Sjögren syndrome, etc.), HIV, and primary immunodeficiencies [57,58,59].

In HIV infection/AIDS, candidosis is an early sign of immunodeficiency and can indicate the immune status and disease progression in such patients [59,60].

Oral candidosis has a wide range of clinical manifestations. Therefore, there is a division into primary candidosis when the infection affects only the oral cavity and perioral area and secondary candidosis when the infection occurs as part of systemic disease. The mucosa is already altered and suitable for infection and lesions associated with *Candida spp.* fungi. According to its clinical features, which include color change, candidosis is often divided into white and red [57,61]. The primary form includes four different conditions: pseudomembranous candidosis, acute erythematous candidosis, chronic erythematous candidosis, and chronic hyperplastic or nodular candidosis [51].

Pseudomembranous candidosis is the most common form and is characterized by white patches or plaques on the oral mucosa that can be easily detached by gentle scraping, because only the upper layer of the mucosal epithelium is infected. The possibility of removal is an accepted differential diagnostic feature that distinguishes this form of candidosis from other white accumulations in the mouth. It typically occurs in neonates (who are likely to become infected through the birth canal), anemic, and immunodeficient individuals (HIV, diabetes, malignancy), patients on topical steroid therapy, and those with xerostomia. Lesions may be localized and generalized, most commonly affecting the tongue, buccal mucosa, soft and hard palate. It is often accompanied by taste disturbances and a bad taste in the mouth [51,57,61,62].

Acute erythematous or atrophic candidosis occurs as a side-effect of systemic therapy with broad-spectrum antibiotics and immunosuppressants and corticosteroids, consequently altering the oral cavity’s flora. It is clinically manifested as a painful red lesion on the dorsum of the tongue, and depapilation of the tongue is often present, with symptoms of burning and taste changes [51,57,61].

Chronic erythematous or atrophic candidosis is also known as denture stomatitis or prosthetic palatitis. It is typical for patients who wear mobile acrylic prosthetic replacements and is most commonly found on the palate in people with total dentures. The onset of the disease is facilitated by poor oral hygiene and inadequate hygiene of the dentures. Lesions on the mucosa are red and limited to areas covered by the prosthetic replacement, sometimes accompanied by a burning sensation, but often are asymptomatic and detected only by dental examination [51,57,61,63,64].

Chronic hyperplastic candidosis is also called *Candida* leukoplakia. Unlike the pseudomembranous form, these white deposits cannot be removed by light scraping. It is characterized by deep infiltration of the oral cavity tissue by the hyphae of the fungus. Most commonly, it is found on the lateral parts of the tongue and buccal mucosa. Clusters may be homogeneous or heterogeneous. Heterogeneous lesions are precancerous conditions because they are a predisposing factor for malignant transformation [51,61].

Among the secondary and other forms of *Candida*-related diseases, it is important to mention angular cheilitis, median rhomboid glossitis, and chronic mucocutaneous candidiasis [51,61].

Angular cheilitis is a disease of multiple etiologies that most often includes anatomical predisposition, xerostomia, immunosuppression, stomatitis caused by prosthetic replacements, and may or may not be associated with existing candidiasis in the mouth. It is an inflammatory condition of one, or more often, both corners of the lips and is clinically manifested by redness, erosions, and crusts that are sometimes covered with white plaque [61,63].

Median rhomboid glossitis is a condition of unknown etiology, often associated with secondary *Candida* infection. The change is located in the middle of the tongue, clinically manifested as a nodular or smooth lesion, and it is asymptomatic [51].

Chronic mucocutaneous candidiasis (CMC) is a heterogeneous group of conditions that reflect the inability to fight off candidial infections. It presents as recurrent, progressive candidiasis of the skin, nails, and mucosae [65]. The oral cavity changes initially resemble pseudomembranous candidosis, while later, they change into a hyperplastic form. It most commonly occurs in childhood and is associated with numerous immune disorders [66]. Defense against *C. albicans* is achieved by both the innate and adaptive host immune systems [67,68]. Research studies, especially CMC patients, have the elucidated mechanisms by which the primary innate and adoptive immune response develops during *C. albicans* infection. It has also been found that susceptibility factors differ significantly between gastrointestinal/vulvovaginal candidiasis and CMC infections. The former are much more dependent on micro-environmental factors, such as local nutrients, pH, bile acids, and local commensal flora. At the same time, genomic sequencing of CMC patients revealed type 3 immunity defects, more specifically in Interleukine-17 (IL-17) pathways [69,70,71]. It was discovered that T helper (Th17) cells have a critical role in immune defense against *Candida*, but also a considerable role against other microbes [72,73]. Up to 80% of patients with childhood-onset CMC develop recurrent or severe infections with organisms other than *Candida*, including cutaneous dermatophyte infections and occasional bacterial septicemia [65,74].

In addition to the mucosa, *Candida spp*. are present on all tooth surfaces–enamel, dentin, and cementum. *C. albicans* grows in enamel cracks and grooves and can penetrate through open dentin tubules [75,76]. Several *Candida spp.* have been isolated from dentin and root caries in children and adults, with a prevalence of 66%–97% in children and 31%–56% in adults [76,77]. *Streptococcus mutans* strains are considered to be the bacteria with the highest cariogenic potential, and in combination with *Candida*, this potential is increased. Bacterial and fungal cells are able to produce glucan, which, as an extracellular polysaccharide, helps create a cariogenic biofilm [76,78]. The presence of *Candida spp*. thus stimulates the growth of *S. mutans* and the volume of the biofilm [79,80]. There is evidence of interaction between *C. albicans* and *Porphyromonas gingivalis*, a bacterium considered the most important in developing periodontitis [81,82]. In a biofilm model, it has been shown that, in the presence of oxygen, *C. albicans* creates a protective environment for *P. gingivalis* [81,83]. To conclude, *Candida* plays a significant role in oral infections.

Treatment of mild to moderate oral infections caused by *C. albicans* usually consists of antifungal drugs applied topically to the inside of the oral cavity for 7 to 14 days. These are the antifungal drugs clotrimazole, miconazole, or nystatin. For severe infections, the antifungal drug fluconazole, taken orally or intravenously, is most commonly used [84].

### 3.2. Candida albicans in Gastroenterology

The gastrointestinal flora is unique in each individual and mostly constant in healthy people. There are differences in its composition along the digestive tract, and it varies from person to person depending on dietary or hygienic habits and age [4,85,86,87]. It is thought that humans are carriers of *C. albicans* by nature, and the gastrointestinal tract is one of the main stocks in the body. This species is the most common opportunistic pathogen among fungi and can cause diseases ranging from superficial to invasive and life-threatening infections [88]. The entry of *C. albicans* into the bloodstream and the development of disseminated candidiasis mostly occur after an invasive infection of the gastrointestinal system [4,89,90]. The main risk factors that promote the transition of *C. albicans* from a commensal or symbiotic to a pathogenic organism are dysbiosis of the residential microbiota, immune dysfunction, and damage to the muco-intestinal barrier [91].

At least one of four events is necessary for disseminated candidosis: direct invasion of epithelial cells (ECs) into blood capillaries and vessels, indirect translocation of *C. albicans* cells phagocytosed by host immune cells, direct damage of mucosal barriers, and spread from fungal biofilms [49,91].

The most common type of infectious esophagitis caused by fungi is esophageal candidosis caused by *C. albicans* [92,93]. The risk of infection exists in immunosuppressed persons (HIV/AIDS-esophagitis caused by *C. albicans* is an AIDS-defining disease, hematological malignancies, transplant patients), and in patients with comorbidities such as diabetes, alcohol consumption and smoking, antibiotic therapy, glucocorticoids, chemotherapy, radiotherapy, upper esophageal damage, and old age [92,93,94,95,96]. The clinical disease may be asymptomatic but is most commonly manifested by acute odynophagia, dysphagia, and pain behind the sternum [94,97]. Endoscopes show plaques of white to light yellow color on the mucosa, which cannot be washed off, and after their removal, the mucosa is red and ulcerative [93,94]. Esophageal candidosis, unlike oropharyngeal, should always be treated with systemic rather than topical antifungals. Three groups of drugs can be used in therapy: nystatin, amphotericin B, and azole antifungals (most commonly fluconazole), where the choice depends on the degree of immunosuppression [93,98]. Esophagitis caused by *C. albicans* is most often superficial, but complications and invasion with hematogenous dissemination (fungemia) are also possible and can subsequently lead to infection of other organs [93].

Fungal diseases of the gastroduodenum are less commonly reported. Mostly they occur as a secondary infection of individuals with tumors in this area, and they infiltrate benign or malignant ulcers that have a reduced ability to heal. Endoscopically, this looks like a white or grayish deposit that separates easily from the mucosa and is located at the base of the ulcer. The ulcer mostly heals with antiulcer therapy [99].

Possible intestinal infection may be superficial when the invasion is limited to the mucosa and submucosa, but can also be deep, where penetration is unlimited, and tissue destruction and perforation of the intestinal wall or spread to distant places occurs. Fungal infections are most commonly associated with inflammatory bowel disease (IBD). Predisposing factors are mucosal damage, mostly caused by surgery and chemotherapy, and impaired neutrophil function due to tumor therapy or long-term glucocorticoid use. When administering TNFα, *C. albicans* should be suspected if infections are detected early during IBD treatment [100].

The interaction of *C. albicans* as a pathogen with the intestinal mucosa occurs in the form of adhesion, invasion, damage, and apoptosis. The main role in infection, and consequently pathogenicity, is played by substances secreted by the fungal hyphae [89,91,101].

Increased colonization and infection increase the secretion of antimicrobial peptides (AMPs) by host cells, but *C. albicans* has developed mechanisms to avoid their activity as the first step in adherence to intestinal epithelial cells (IEC). In addition to defending against AMPs, *C. albicans* must break down the mucus’s protective layer to reach the epithelial cell layer. After adhering to the mucins, it secretes mucinolytic enzymes. After the first contact with the IEC, most fungal cells convert to the hyphal form and express genes that promote adhesion by releasing adhesins and hyphal invasions. The release of surface molecules, i.e., adhesins, is crucial in the process of adhesion to the host tissue. It can also adhere to enterocytes via polysaccharide molecules on the cell wall surface [4,102,103,104].

Invasion by *C. albicans* takes place through two mechanisms, namely endocytosis and active penetration. Endocytosis is a host-driven process that does not require sustainable hyphae and occurs in the first four hours of interaction. Active penetration into the IEC is a procedure that requires sustainable forms of the fungus but does not require host activity and depends on the type of epithelial cells. It is thought that penetration takes place by the combination of mechanical pressure created by progressive elongation of the hyphae and lytic activity. This procedure allows *C. albicans* entry into enterocytes while the intercellular junctions remain intact. Unlike oral cells, fungi need specific genes to invade enterocytes [4,91].

Invasion of *C. albicans* fungi contributes to IEC damage and cell death. It is assumed that necrotic cell death is the main mechanism of damage observed during translocation of *C. albicans* through enterocytes. After the hyphae form, a cytolytic toxin called candidalysin is secreted, which is responsible for damaging host cells. It is the first peptide toxin identified in human fungal pathogens [4].

Antifungal therapy should be utilized in patients with intra-abdominal infections and risk factors for candidiasis, such as recent abdominal surgery, necrotizing pancreatitis, or anastomotic leakage. Additionally, infection source control should be included. The choice of antifungal therapy is similar to that for the treatment of candidaemia. An echinocandin is recommended as the initial therapy, while fluconazole is an alternative therapy. Azoles are used if the infection is not caused by *Candida* species resistant to them [105].

### 3.3. Candida albicans in Dermatovenerology

*Candida* spp. infections are one of the most common fungal infections in dermatology. *Candida albicans* is responsible for 80–90% of infections, but other *Candida* species are frequently seen as causative pathogens [74]. *Candida* infections are considered opportunistic in the majority of cases because *Candida albicans* is a normally commensal fungus. However, when host-immunity is impaired for various possible reasons, a pathogenic infection may occur [67]. Overall, a balance between the host defense system and the virulence factors of *Candida albicans* is the key to the commensal relationship. *Candida albicans* usually causes superficial skin infections, while “deep” mycoses, with the dermis and subcutis’ involvement due to *Candida*, are rare. However, in severely immunocompromised patients, an invasive fungal infection may occur, resulting in deep penetration and systemic candidiasis, often with a fatal outcome.

Mucocutaneous candidiasis has a wide spectrum of clinical presentations. They depend on the affected body site, the patient’s age group, and various predisposing factors. The unifying clinically relevant features of candidiasis are erythema, erosions, and easily removed cheesy white plaques. While white plaques are more often seen on mucosa than on skin, erythema and erosions are widely unspecific presentations. Therefore, taking a good anamnesis with special attention to the patient’s potential predisposing factors is crucial for a dermatologist to establish a clinical suspicion of candidiasis. When typical body regions are affected, this is also a clue for a successful diagnosis.

The most common sites of involvement are intertriginous zones, e.g., submammary, inguinal folds, intergluteal creases, and pannus folds in overweight patients. *Candida* infections are present with sharply marked erythematous, sometimes erosive patches with light scaling of the surface, and satellite papules and pustules on the periphery [106]. These pustules are generally sterile and are called spongiform pustules due to the collections of neutrophils within the epidermis [74].

*Candida* also can cause chronic and acute paronychia and onychomycosis. Paronychia is inflammation of the skin around a nail (periungual area), also called whitlow, and is usually due to bacterial infection with *Staphylococcus aureus* and *Pseudomonas* spp. Onychomycosis is a fungal nail infection, and when caused by *Candida*, it is usually secondary to chronic paronychia [107].

Erosio interdigitalis blastomycetica (EIB) is another common *Candida* infection of the web space between the third and fourth fingers, usually in patients whose hands are frequently in water. It is assumed that this space is at least mobile, so the retention of water, sweat, and other potential irritative agents, such as soap, is most likely creating a perfect base for *Candida* infection. Typically, there is a central erosion surrounded by a rim of white macerated skin [108].

In rare cases, *Candida albicans* may cause folliculitis, usually of the beard area in an adult man with severe immunosuppression. In such cases, it can involve deep layers of skin with a clinical finding of deeper nodules around the hairs and pustules.

The genital area in men and women is a frequent site of *Candida* infection. Approximately 15–30% of asymptomatic women are vaginal carriers of *Candida spp*.; during pregnancy, this rises to 40% [109]. It is assumed that almost every woman has at least one incident of vulvovaginal candidiasis in her lifetime. In men, balanitis and balanoposthitis are seen. In both sexes, the involvement of the perianal area is also possible. Even without clinical findings on the skin, localized pruritus of the genital and perianal area alone can be a sign of candidiasis [110].

Another presentation of *Candida* infection is diaper dermatitis. Diaper dermatitis is inflammation of the skin under the diaper, commonly seen in infants but also in incontinent adults. It is often a form of irritant contact dermatitis, but many cases are complicated with *Candida* superinfection. Diaper dermatitis is the cumulative result of several factors that damage the normal skin barrier. At first, occlusion that leads to skin maceration and skin contact with urine was suggested as the main reasons, but today fecal bacteria and the alkaline pH of urine are considered to have a greater role [74].

The skin of the newborns differs from that of adults in several ways. The possibility of *Candida* to produce disseminated cutaneous infection and systemic infection is thus higher, especially in premature newborns [111]. Congenital candidiasis represents an intrauterine infection. Extensive areas of erythema, papules, and pustules can be seen, as well as diffuse “burn-like” erythema, with desquamation and erosions [112]. Neonatal candidiasis is acquired during delivery or postnatally and is more similar to the adult presentation, involving more typical sites such as the diaper and intertriginous areas [113].

Besides earlier mentioned CMC, hyperimmunoglobulin E syndrome (HIES) is another group of inherited disorders characterized, among other things, by recurrent cutaneous candidiasis, occurring in approximately 80% of HIES patients [114,115]. Mutations in the *STAT3* (signal transducer and activator of transcription 3) encoding gene cause the classic form of HIES, so susceptibility to *Candida* infections reveals the important role of STAT3-dependent cytokines (e.g., IL-23, IL-21, IL-6) in the differentiation of IL-17 producing T helper cells [116].

Besides hereditary defects in host immunity mechanisms, numerous external factors contribute to candidial infections. As mentioned earlier, they are commonly seen in patients with diabetes mellitus [117,118,119], immunosuppressed patients, such as HIV/AIDS patients, oncology patients receiving chemotherapy, and organ transplant patients [117,120,121] taking broad-spectrum systemic antibiotics [122,123]. In modern medicine, especially with emerging novel therapies, the number of such patients is growing, so *Candida* infections are also increased [68]. For example, remarkable advances are taking place in the treatment of immune-mediated inflammatory diseases (IMID). One of the important specific therapeutic targets is cytokine IL-17 because it plays a critical role in the pathogenesis of various IMIDs, including psoriasis [124,125,126]. Dermatologists working with patients receiving these therapies should warn them about the increased risk of candidiasis. As mentioned before, IL-17 pathways play a critical role in the host defense against *Candida*, so blocking them will obviously result in greater susceptibility to infection [126,127].

The strategy for treating cutaneous candidiasis depends on the infection’s location, its extensiveness, and the patient’s immune status. Most usually respond to topical antifungal agents, but if the patient is immunocompromised, has extensive areas affected, or fails to respond to topical therapy, systemic treatment is needed [128]. Although many antifungal agents are available, not all of them are effective against *Candida* [105]. Limited effectiveness is not the only therapeutic challenge. Today, there is increasing pathogen resistance to antifungal agents, and *Candida* species are among them [67,129]. Generally, mucocutaneous candidiasis responds well to topical azoles (miconazole, clotrimazole, and econazole) [74,105,130]. Of the topical polyenes, nystatin is most commonly used, especially for oral and vulvovaginal candidiasis [105,131]. An antifungal agent that is more effective against *Candida* than the azoles, allylamines, or benzylamines is ciclopirox olamine. It is increasingly being used in dermatology practice [132,133]. If systemic treatment is necessary, fluconazole is generally the drug of choice [105]. However, recently azole-resistant strains have been detected [134]. Alternative choices are itraconazole or one of the second-generation triazoles available. If the patient is not responding to treatment with azoles, then amphotericin B therapy should be tried. Members of the echinocandin class of antifungal drugs (micafungin, anidulafungin, caspofungin acetate) also showed effectiveness in *Candida* infections [105,135]. Until recently, ketoconazole was also commonly used, but the EMA and FDA have withdrawn it due to potential hepatotoxicity, and today its use is restricted to only complicated, non-responsive fungal infections [136]. Its systemic use is limited in several countries, although it is still being used locally (as shampoo).

The most common side effect of topical antifungals is local skin irritation, but rarely true allergic contact dermatitis. Systemic antifungal drugs may cause severe cutaneous reactions, including toxic epidermal necrolysis and Stevens-Johnson syndrome. Fluconazole can cause potential hepatic damage. The main mechanism of hepatotoxicity is drug-drug interactions caused by inhibition of cytochrome P450 enzymes, which play a major role in the metabolism of lipophilic drugs. All systemic antifungals should be used with caution in patients with renal or liver disease [137].

To conclude, it is worth mentioning traditional topical medications, used additionally in the treatment of cutaneous candidiasis: potassium permanganate (KMnO_4_), gentian violet solution, Burow’s solution (aluminum acetate) [74]. Additionally, in the search for novel antifungal preparations, the use of silver has reemerged [138]. Recently, the effect of silver nanoparticles (AgNPs) on various dermatophytes and yeasts, including *Candida spp*. was tested, and many studies showed that AgNPs have potential in the treatment of candidiasis [139,140].

### 3.4. Invasive Candida albicans Infections

Invasive candidiasis refers to bloodstream infections caused by *Candida spp.* Most often occur after passing through the intestinal barrier. For example, after surgery, it could spread to the abdominal cavity, and enter the bloodstream and cause candidiasis [141]. *C. albicans* overgrowth can trigger an impairment of the immune response, leading to opportunistic infections in various organs, i.e., invasive candidiasis [142]. Therefore, the invasive disease is usually the result of increased or abnormal colonization, along with local or generalized lack of host defense [143]. Invasive candidiasis is not a single clinical entity but is a disorder with countless clinical manifestations that can potentially affect any organ [141] (Table 1). Invasive candidiasis is closely related to medical technology development and is widely recognized as a significant cause of morbidity and mortality in the health care environment [144]. At least 15 different *Candida spp.* can cause infections in humans. However, five pathogens cause the most invasive infections: *Candida albicans*, *Candida glabrata*, *Candida tropicalis*, *Candida parapsilosis*, and *Candida krusei*, but the most common pathogen in the clinical setting is *C. albicans* [141].

Invasive *Candida* infections are most often associated with candidemia (*Candida* species in the blood), primarily in immunosuppressed patients and those requiring intensive care. Immunosuppressed patients are at special risk for candidemia, including those with hematological malignancies (in patients who have just recovered from an episode of neutropenia), recipients of hematopoietic cell transplants or solid organ, and those given chemotherapeutic agents for a variety of different diseases. Other risk factors are associated with extensive gastrointestinal mucosal damage, broad-spectrum antibiotics, and central venous catheters. *Candida albicans* is the most typical cause of candidiasis, although many *Candida* species other than albicans have been isolated in recent years [105,145]. When *C. albicans* enters the bloodstream, it can cause infections of the bladder and kidneys, endophthalmitis, meningitis, osteoarticular infections, endocarditis, peritonitis and intra-abdominal infections, pneumonia, empyema, mediastinitis, and pericarditis.

Candiduria refers to the presence of *Candida* in the urine. Candiduria as a source of candidemia typically occurs in patients who have urinary tract abnormalities, most often urinary tract obstruction, and/or in those who have undergone a urinary tract procedure. It is common in hospitalized patients, although it is often challenging to distinguish colonization from a bladder infection. Renal transplantation was thought to be a risk factor for ascending infection and candidemia when candiduria was present. Studies show that more than 50% of hospitalized patients with candiduria have *Candida albicans* isolated [146,147].

*C. albicans* does not often cause clinically meaningful pneumonia in adults. Despite that, *Candida albicans* is often isolated from patients’ respiratory tracts in intensive care units, intubated patients, or patients with a chronic tracheostomy. In most cases, this reflects colonization of the airways and not an infection [105]. *Candida* pneumonia has been noted in seriously immunocompromised patients with disseminated conditions, deficient birth weight newborns, and individuals with malignancies [148,149,150]. Since contamination issues confuse an antemortem diagnosis, a final diagnosis of invasive *Candida* pneumonia needs histological verification, which is typically achieved only at autopsy. A bronchoalveolar lavage is a diagnostic tool for verifying pneumonia and determining the causative pathogen [151].

*Candida* species infect bones and joints due to either hematogenous seeding or inoculation during trauma, intra-articular injection, a surgical procedure, or injection drug use. Osteoarticular infections often become symptomatic months or as long as a year after an episode of fungemia or a surgical procedure. The manifestations are generally more subtle than bacterial infections at the same sites. Both of these factors contribute to long delays in diagnosis, especially in patients with vertebral osteomyelitis. The major symptoms of *Candida* arthritis are pain and decreased range of motion, whereas local pain is the predominant symptom of *Candida* osteomyelitis. Only one *Candida* colony is considered pathogenic in a biopsy or aspirate culture of joint fluid or bone [152,153,154].

*Candida* infections of the central nervous system most typically affect the meninges (although they are all generally uncommon). This most often happens in premature infants. The infection may be secondary to hematogenous spread or direct inoculation. Predisposing factors include neurosurgery, newer antibiotics, and corticosteroids. Fever, meningismus, elevated cerebrospinal fluid pressure, and localizing neurological signs are often present. *Candida albicans* seems to be the most pathogenic *Candida* *spp*., leading to increased mortality rates in invasive infection when compared to other *Candida* species [155,156].

Fungal endocarditis represents 1–6% of the total spectrum of endocarditis. *Candida* endocarditis is one of the most severe candidiasis manifestations and is the most common cause of fungal endocarditis [157]. Due to the rarity of candidal infective endocarditis, the prognosis, epidemiology, and optimal treatment of *Candida* infective endocarditis have been insufficiently described. Therapy procedures are obtained mainly from single-site case series and case reports. *Candida* endocarditis results from candidemia and is usually seen in patients with prosthetic heart valves, people who inject intravenous drugs, and in patients who have indwelling central venous catheters and prolonged fungemia [158].

*Candida albicans* (and other yeasts) can cause nosocomial infections, which involve the transmission by the hands of healthcare professionals or contaminated material (e.g., rinsing the central venous catheter with saline used for multiple patients) [141,159]. Critical challenges in treating candidaemia and invasive candidiasis include prevention, early detection, and rapid initiation of appropriate systemic antifungal therapy. The untimely initiation of treatment could result in poorer clinical outcomes [160,161]. The emergence of antifungal resistance is a new problem globally, which is an additional concern in treating infections caused by *C. albicans* and other *Candida sp.* [162,163,164]. Published data suggest that fluconazole antifungal prophylaxis reduces the incidence of invasive candidiasis among high-risk ICU patients [165]. However, it should be borne in mind that antifungal prophylaxis may favor resistance development [163]. Targeted antifungal prophylaxis is warranted in high-risk recipients of the liver, pancreas, small intestine, or hematopoietic stem cells [166]. The control of the infection source and early initiation of treatment with effective systemic antifungal therapy, usually before the diagnosis of invasive candidiasis is confirmed, is crucial for successfully treating invasive candidiasis [141,167]. Source control refers to removing the infection source, such as removing contaminated intravascular catheters, drainage of peritoneal fluid, pleural fluid, or abscesses [12,168]. To successfully treat the infection, it is sometimes necessary to remove infected prosthetic devices, such as a pacemaker, an artificial joint, or other prosthetic devices, if possible [169]. In addition to controlling the source of infection, early effective antifungal therapy is crucial in successfully treating patients with invasive candidiasis. The data indicate significantly higher mortality when antifungal therapy is delayed or inadequate or when rapid source control has not been achieved [105,141,170].

Several published guidelines outline expert recommendations for treating invasive candidiasis, with detailed recommendations for specific clinical circumstances [105,171,172,173] (Table 2). The selection of an antifungal drug for initial treatment should be based on prior testing of an isolated strain of *C. albicans* for antifungal drugs, the patient’s sensitivity to the antifungal agent, and the severity of the disease, relevant comorbidities, and brain involvement, heart valves, or internal organs [174,175]. Specific clinical units should also consider data on susceptibility to *Candida spp.* infections.

## 4. Conclusions

It is extremely important to know the factors and mechanisms of the pathogenicity of *C. albicans* precisely because of their wide range, from dimorphism, biofilm formation, thigmotropism, expression of adhesion proteins, and secretion of extracellular hydrolytic enzymes. *C. albicans* is able to cause infections ranging from superficial to systemic and life-threatening.

In addition to knowing the virulence factors, knowledge of the essential predisposing factors for the development of candidiasis is also necessary, such as neutropenia, immunosuppression, diabetes, age, as well as factors related to patient care, long-term antimicrobial therapy, long-term hospitalization, catheter use, and surgery.

As knowledge of all these factors increases, the possibility of prevention increases, as we work to prevent the occurrence of infections caused by *C. albicans*, and opportunities are created to develop new diagnostic and therapeutic possibilities.

## Figures and Tables

**Table 1:** Invasive candidiasis in various organs.

Bone	Brain	Eye	Heart	Kidney	Liver and Spleen	Lung
Osteomyelitis	Brain abscess	Choroiditis	Endocarditis	Candiduria	Chronic disseminated candidiasis	Focal abscess
Spondylodiscitis	Meningo-encephalitis	Retinitis		Pyelonephritis	Focal abscess	
		Endophthalmitis		Pyonephrosis		
				Renal abscess		

**Table 2:** The recommended treatments of invasive candidiasis.

*Candida albicans*		
Preferred Initial Therapy	Alternative Initial Therapy	Preferred Step-Down Therapy
Echinocandin	Fluconazole	Fluconazole
